# Radiomics nomogram based on dual-energy spectral CT imaging to diagnose low bone mineral density

**DOI:** 10.1186/s12891-022-05389-4

**Published:** 2022-05-06

**Authors:** Qianqian Yao, Mengke Liu, Kemei Yuan, Yue Xin, Xiaoqian Qiu, Xiuzhu Zheng, Changqin Li, Shaofeng Duan, Jian Qin

**Affiliations:** 1 Department of Radiology, The Second Affiliated Hospital of Shandong First Medical University, No.366 Taishan Street, Taian, 271000 Shandong China; 2 GE Healthcare, Pudong new town, No1, Huatuo road, Shanghai, 210000 China

**Keywords:** Osteoporosis, Radiomics, Dual-energy spectral CT

## Abstract

**Background:**

Osteoporosis is associated with a decrease of bone mineralized component as well as a increase of bone marrow fat. At present, there are few studies using radiomics nomogram based fat-water material decomposition (MD) images of dual-energy spectral CT as an evaluation method of abnormally low Bone Mineral Density (BMD). This study aims to establish and validate a radiomics nomogram based the fat-water imaging of dual-energy spectral CT in diagnosing low BMD.

**Methods:**

Ninety-five patients who underwent dual-energy spectral CT included T11-L2 and dual x-ray absorptiometry (DXA) were collected. The patients were divided into two groups according to T-score, normal BMD(T ≥ -1) and abnormally low BMD (T < -1). Radiomic features were selected from fat-water imaging of the dual-energy spectral CT. Radscore was calculated by summing the selected features weighted by their coefficients. A nomogram combining the radiomics signature and significant clinical variables was built. The ROC curve was performed to evaluate the performance of the model. Finally, we used decision curve analysis (DCA) to evaluate the clinical usefulness of the model.

**Results:**

Five radiomic features based on fat-water imaging of dual-energy spectral CT were constructed to distinguish abnormally low BMD from normal BMD, and its differential performance was high with an area under the curve (AUC) of 0.95 (95% CI, 0.89–1.00) in the training cohort and 0.97 (95% CI, 0.91–1.00) in the test cohort. The radiomics nomogram showed excellent differential ability with AUC of 0.96 (95%CI, 0.91–1.00) in the training cohort and 0.98 (95%CI, 0.93–1.00) in the test cohort, which performed better than the radiomics model and clinics model only. The DCA showed that the radiomics nomogram had a higher benefit in differentiating abnormally low BMD from normal BMD than the clinical model alone.

**Conclusion:**

The radiomics nomogram incorporated radiomics features and clinical factor based the fat-water imaging of dual-energy spectral CT may serve as an efficient tool to identify abnormally low BMD from normal BMD well.

## Introduction

Osteoporosis is a systemic skeletal disease characterized by bone loss leading to an increased risk of fragility fractures [1]. With the aging of the population, the incidence of osteoporosis and fragility fractures is increasing. If not treated in time, these fractures would lead to high morbidity and mortality and carries social and economic burdens [2–4]. Therefore, it is very important to early detect osteoporosis, and reduce the incidence of fracture complications.

At present, dual x-ray absorptiometry (DXA) is the most widely used screening tool for osteoporosis and preferred method for bone mineral density (BMD) measurement [5]. According to WHO guidelines [1], a DXA-derived T- score less than − 1.0 indicates an abnormally low BMD, and when the T-score is greater than − 1.0 is normal.

The recently introduced dual-energy spectral CT, which is based on a single tube fast switching between low-energy (80 kV) and high-energy (140 kV) data sets, provides precisely material decomposition (MD) images (e.g. fat–water-based and iodine–water-based MD images) [6]. The dual-energy spectral CT has been used clinically to quantitatively estimate calcium concentration in trabecular bone and cortical bone in patients under going hemodialysis with secondary hyperparathyroidism [7] and to differentiate small hepatic hemangioma from small hepatocellular carcinoma [8]. Osteoporosis is associated with a decrease of bone mineralized component as well as a increase of bone marrow fat, which is caused by a shift of differentiation of mesenchymal stem cells to adipocytes [9]. Fat-water-based MD images of dual-energy spectral CT can reflect the changes of fat content in the process of osteoporosis. However, there are few studies using fat-water MD images of dual-energy spectral CT as an evaluation method of osteoporosis.

The term radiomics has attracted increased attention in recent years, and it is a promising technique using computerized quantitative imaging analysis to extract an enormous quantity of image-related features, followed by subsequent data analysis for decision support [10–13]. Radiomics nomogram combined the fat-water imaging of dual-energy spectral CT can provide a new and effective method for clinical and radiologists to quantify bone marrow fat in the process of osteoporosis. Therefore, the purpose of this study is to establish and validate a radiomics-clinical model (radiomics nomogram) that combined the fat-water imaging of dual-energy spectral CT based radiomics signature and clinical risk factors for discriminating abnormally low BMD from normal BMD.

## Materials and methods

### Patients

This retrospective study of opportunistic screening was approved by the institutional review board, and the requirement to obtain informed consent was waved. The primary cohort of this study was identified by searching the institutional picture archiving and communication systems (PACS) database for medical records from January 2020 to August 2020 to identify patients with DXA as well as dual-energy spectral CT examination for other indications in routine practice. For inclusion, patients had to have had thoracic or abdominal dual-energy spectral CT that showed T11-L2 were included in the study, or both, as well as DXA of the hips and lumbar spine. The mean time interval between the two examinations was less than 1 week. Exclusion criteria included image artifacts obscuring the spine, or any hardware or metal associated with the spine, as well as patients with spinal fractures, spinal tumors, endocrine diseases, rheumatic diseases, and infectious spondylitis, because these diseases are known to cause osteoporosis. Baseline clinic data, including the values of T11-L2 of fat-water images, age, gender, body mass index (BMI) derived from medical records were also recorded. The final cohort consisted of 95 patients (33 men, 62 women; mean age, 61.69 ± 9.30 years).

### DXA examination and diagnostic criteria of osteoporosis

DXA of the lumbar spine and proximal femora was performed for BMD assessment by using standard techniques according to manufacturer and WHO guidelines Hologicdiscovery dual-energy X-ray bone densitometer (Hologic Inc., Bedford, MA) [1]. According to WHO guidelines, a DXA-derived T-score less than − 1.0 indicates an abnormally low BMD, which is further categorized into osteopenia (T-score between − 1.0 and − 2.5) and osteoporosis (T-score of − 2.5 or below), and when the T-score is greater than − 1.0 is normal. In our study, the patients were divided into two groups according to T-score, normal BMD(T ≥ -1) and abnormally low BMD(T<-1).

### Dual-energy spectral CT examination

All patients underwent routine thoracic or abdominal dual-energy spectral CT with a scanner (Revolution CT; GE Healthcare, Wauwatosa, Wis) and a single-tube, fast dual-tube potential (80 kVp and 140 kVp) switching scan technique. Imaging parameters were as follows: tube voltage = 80/140KV; pitch = 0.984; rotation time = 0.8 s/r; slice thickness = 1.25 mm, slice interval = 5 mm, adaptive statistical iterative reconstruction V (Asir-V) = 40%.

### Image segmentation and feature extraction

**Fig. 1:**
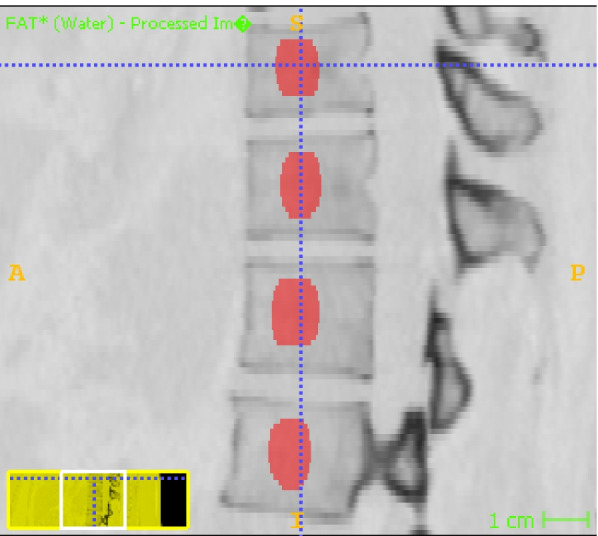
An ovoid region of interest (ROI) of T11-L2 was manually drawn on the sagittal images with the same size, typically selecting a representative trabecular level centered between this inferior and the superior endplate

The material decomposition images with fat-water as base materials obtained from the dual-energy spectral CT imaging were transferred to ITK-SNAP software (Version 3.6.0, www.itksnap.org) for segmentation. An ovoid region of interest (ROI) of T11-L2 was manually drawn on the sagittal images with the same size, typically selecting a representative trabecular level centered between this inferior and the superior endplate (Fig. 1). Care was taken to avoid the posterior venous plexus, focal heterogeneity, cortical bone or any imaging-related artifacts. To assess the segmentation availability, the image segmentation was examined by another radiologist with 10 years of experience in CT interpretation. If the ROI was questioned, it would be re-segmented after the two agree.

AK software (AnalysisKit, version 3.2.0, GE Healthcare, China) backend with pyradiomics (version 3.0.1, https://pyradiomics.readthedocs.io/en/latest/) was used to extract the radiomics features. Before feature extraction, the images were preprocessed with 3 steps, resampling the voxel size into 1*1*1 mm^3, discretizing the gray values using 25 bin width, normalizing the gray value using *μ* ± 3*σ* method. After that, 6 classes of features were extracted, first-order features, gray level co-occurrence matrix features, gray level run length matrix features, gray level size zone matrix features, neighboring gray tone difference matrix features and gray level dependence matrix features, based on the original image, wavelet-transformed images and Laplacian of gaussian filtered images with sigma 2, 3. Totally, 828 features were extracted and used in the following analysis.

### Data preprocessing

The dataset was randomly assigned in a 7:3 ratio to either the training cohort or test cohort. All cases in the training cohort were used to train the predictive model, while cases in the test cohorts were used to independently evaluate the model’s performance.

Before analyses, variables with zero variance were excluded from analyses. Then, the missing values and outlier values were replaced by the median. Finally, the data were standardized using z-score method.

### Development of radiomics signature

We used two feature selection methods, the minimum-Redundancy Maximum-Relevancy (mRMR) and the least absolute shrinkage and selection operator (LASSO) to select the feature. At first, mRMR was performed to eliminate the redundant and irrelevant features, 30 features were retained. Then LASSO was conducted to choose the optimized subset of features to construct the final model. Radscore was calculated by summing the selected features weighted by their coefficients.

### Development of radiomics nomogram and assessment the performance of different models

The clinical variables included the values of T11-L2 of fat-water images, age, gender and BMI. We used the univariate logistic regression analyses to filter these variables and select the significant risk factors with *P* < 0.05, subsequently, a backward step-wised multivariable logistic regression analysis with Akaike An Information (AIC) as criterion was performed to construct the clinical model. Meanwhile, a radiomics nomogram combining the final radiomics signature and independent clinical risk factors were built. Here, we used DeLong’s test to compare whether the ROC curves were different between nomogram and clinical model. The calibration of the nomogram was evaluated with a calibration curve analysis and using LOESS method. The goodness of fit was tested using the Hosmer-Lemeshow test. Finally, we used decision curve to evaluate the clinical utility of the model.

### Statistical analysis

All statistical analyses for the present study were performed with R (version 4.0.2, www.r-project.org). The mRMR and LASSO were used to select the features and were performed using ‘mRMRe’ and ‘glmnet’ packages, respectively. The LASSO includes choosing the regular parameter λ, determining the number of the feature. Wilcoxon test was applied to compared the radscores from abnormal low BMD group and normal BMD group on training cohort and test cohort respectively. Receiver operating characteristic (ROC) curve analysis was performed to evaluate the performance of the model, and area under curve (AUC) were calculated. Besides, DeLong’s test was used to compare whether the ROC curves were different between nomogram and clinical model, we also used net reclassification index (NRI) and integrated discrimination improvement (IDI) to compare their accuracy and discrimination ability. A two-tailed *p*-value < 0.05 indicated statistical significance.

## Results

### Patients characteristics

A total of 95 patients were included in our study. There were 67 and 28 patients in the training and test cohort respectively. The detailed patient characteristics in the two cohorts are displayed in Table 1. In each cohort, there was no significant differences in gender and age, but differences of the values of fat-water imaging sets were both detected in the two cohorts between the normal BMD patents and abnormally low BMD patents(*P* = 0.000). BMI was not statistically significant in the test cohort, however, it was statistically significant in the training cohort.

**Table 1:** Characteristics of patients in the training and test cohorts

Characteristics	Training cohort(*n* = 67)			Test cohort(*n* = 28)		
	Normal BMD	Abnormally low BMD	*P*-values	Normal BMD	Abnormally low BMD	*P*-values
Number	21	46		9	19	
Gender(%)						
Male	10 (47.6)	12 (26.1)		4 (44.4)	7 (36.8)	
Female	11 (52.4)	34 (73.9)	0.144	5 (55.6)	12 (63.2)	1.000
Age, y, mean ± SD	61.8 ± 9.4	61.6 ± 9.0	0.933	57.9 ± 10.8	63.6 ± 9.2	0.1479
BMI, mean ± SD	25.8 ± 2.3	23.9 ± 3.5	0.021*	26 ± 2.9	24.4 ± 2.6	0.1467
Values of fat-water imaging, mean ± SD	− 3906.4 ± 763.8	− 2590.3 ± 718.4	0.000*	− 4491.3 ± 1184.3	− 2581.2 ± 603.3	0.000*

*P value < 0.05, two-sample t-test for continues variables; χ^2^ test and Fisher’s exact test for categorized variables. *BMD* Bone mineral density, *BMI* Body mass index, *SD* Standard deviation

### Feature selection

**Fig. 2:**
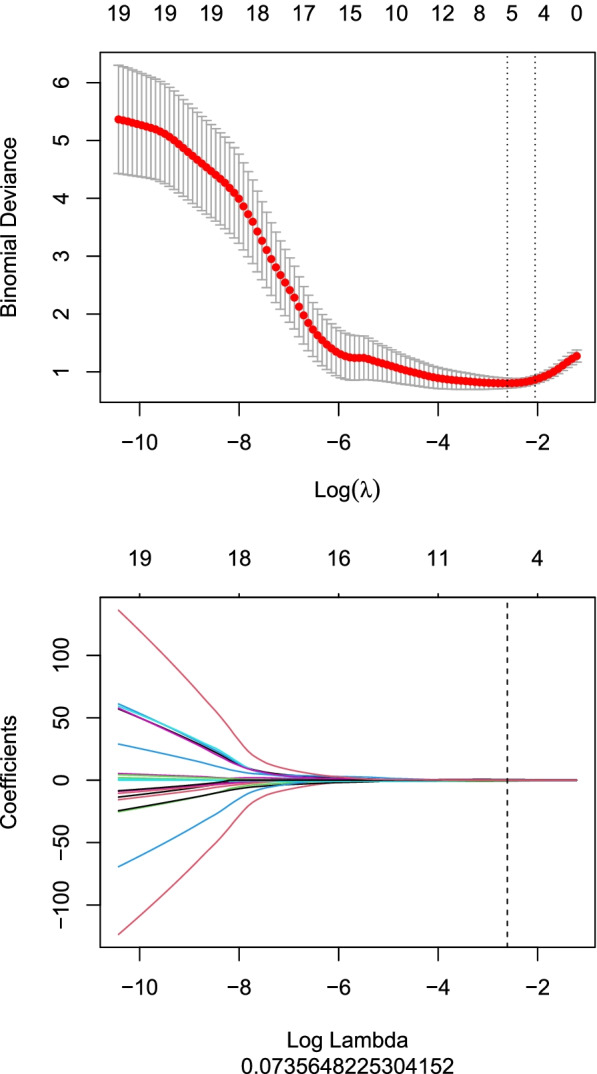
Selection of the hyperparameter (λ) in the LASSO model via 10-fold cross validation based on minimum criteria of binomial deviance. Binomial deviance was plotted as a function of log (λ). The optimal λ value of 0.073 was selected. The dotted vertical line was drawn at the value selected using 10-fold cross-validation, in which the selected λ resulted in 5 non-zero coefficients. LASSO:the least absolute shrinkage and selection operator

**Fig. 3:**
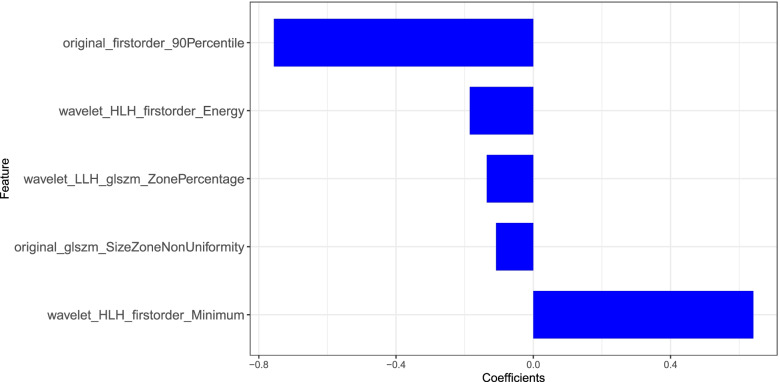
The final 5 features extracted from fat-water imaging of dual-energy spectral CT and their coefficients after LASSO. LASSO:the least absolute shrinkage and selection operator

Eight hundred twenty-eight features were finally extracted from one image. Due to none of features was 0 variance, 0 feature was removed. So that, 828 features were all imported into mRMR feature selection, which was performed to eliminate the redundant and irrelevant features, 30 features were retained. Then LASSO was conducted to choose the optimized subset of features to construct the final model (Fig. 2), the specific steps included: using 10-fold cross validation to find the optimized hyperparameter λ with minimum binomial deviance as criteria, and then determining the optimized feature subset according to the λ. Finally, 25 features were removed and five features were selected as the most predictive subset of feature, which and their corresponding coefficients were shown in Fig. 3.

### Radiomics signature construction

Radscore was calculated by summing the selected five features weighted by their coefficients. The final formula of radscore is: Radscore = − 0.136*wavelet_LLH_glszm_ZonePercentage-0.756*original_firstorder_90Percentile-0.185*wavelet_HLH_firstorder_Energy+ 0.641*wavelet_HLH_firstorder_Minimum-0.109*original_glszm_SizeZoneNonUniformity + 1.022.

**Fig. 4:**
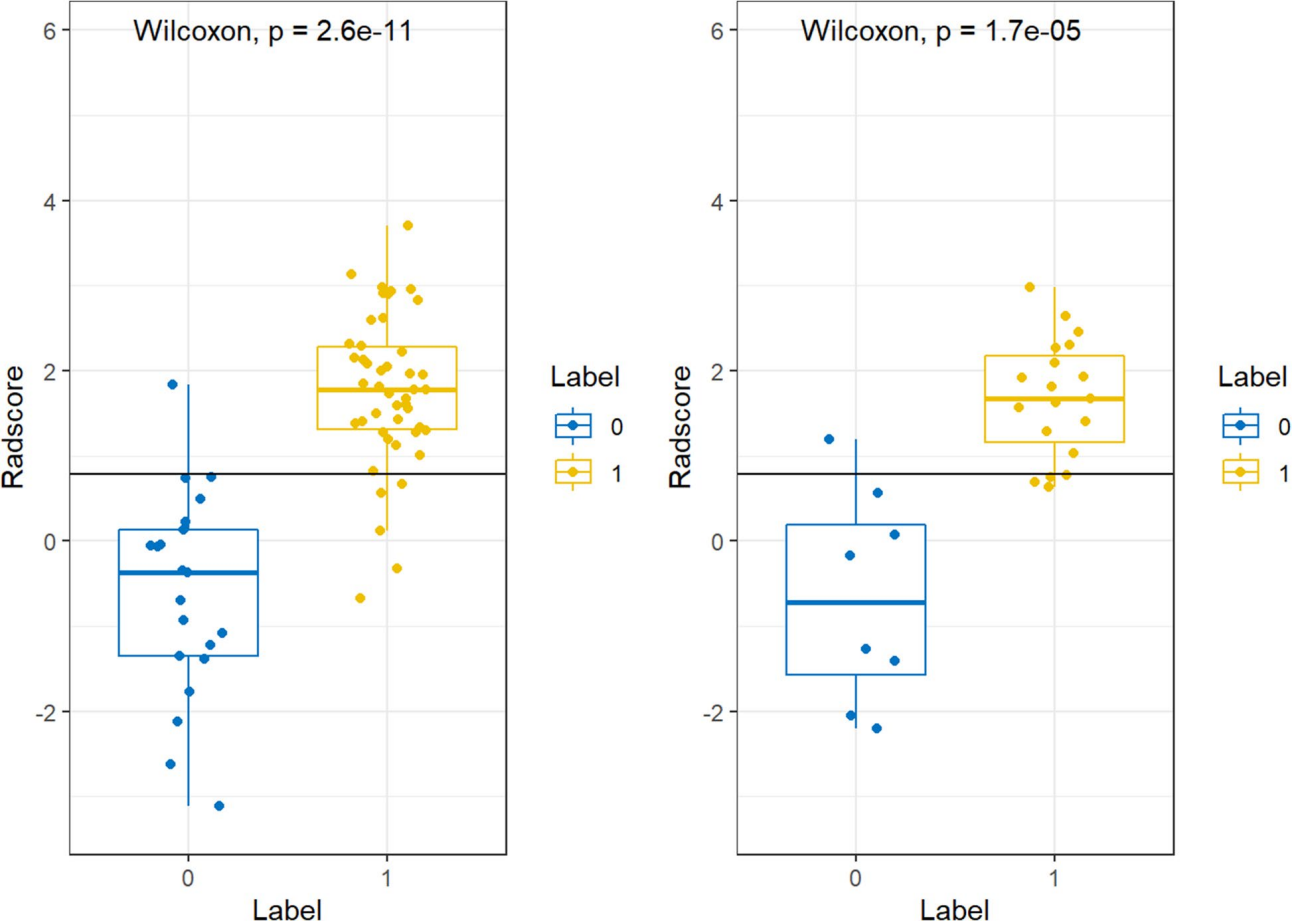
Boxplots show the dual-energy spectral CT radiomics signatures in abnormal BMD patients were much higher than the normal BMD group in both the training (left) and test cohort (right). 0 means normal BMD group, 1 means abnormal BMD group

And we compared the radscores from normal BMD and abnormally low BMD of the fat-water imaging on training and test cohort respectively. Boxplots show the dual-energy spectral CT radiomics signatures in abnormally low BMD patients were much higher than the normal BMD group in both the training and test cohort (Fig. 4). The AUC values of the radiomics model in the two cohorts were 0.95 (95% Confidence interval [CI]: 0.89–1.00, *P*<0.001), 0.97 (95% CI: 0.91–1.00, *P*<0.001) respectively, indicating that radiomics features could effectively distinguish abnormally low BMD from normal BMD.

### Nomogram build

**Fig. 5:**
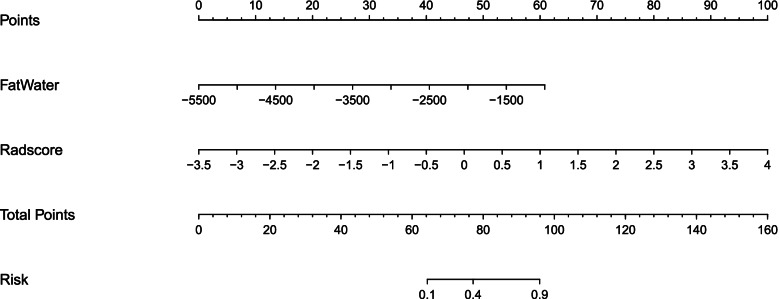
The radiomics nomogram, combining the values of fat-water imaging and radscores developed in the training cohort

By incorporating the values of fat-water imaging and radscores, a radiomics nomogram was developed in the training cohort (Fig. 5) using the following formula: Nomoscore = (Intercept)*5.693 + FatWater*0.0019 + Radscore*1.855.

**Fig. 6:**
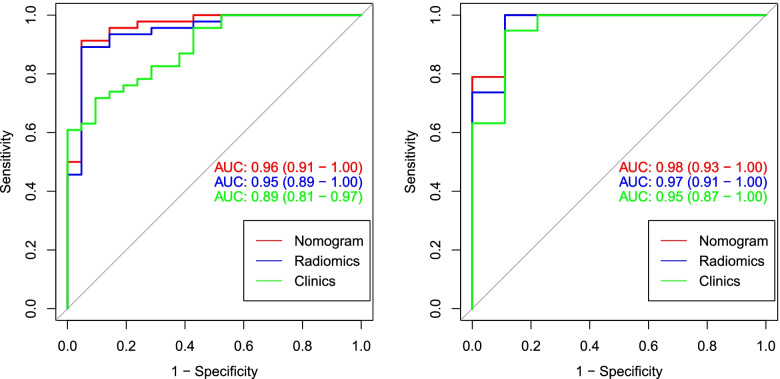
Comparison of ROC curves among the clinical, radiomic and radiomics nomogram model for the prediction of BMD normal or abnormal in the training (left) and test (right) cohort

The ROC of radiomics nomogram and clinical factors model are exhibited in Fig. 6. Based on the radiomics nomogram and clinics model, the AUC values for training cohort were 0.96(95% CI: 0.91–1.00), 0.89(95% CI: 0.81–0.97), respectively; and the AUC values for test cohort were 0.98(95% CI: 0.93–1.00), 0.95(95% CI: 0.87–1.00), respectively. The diagnostic performance of every model is demonstrated in Table 2. The nomogram had better accuracy and discrimination abilities than clinical model in both training and test cohort (Training group: nomogram vs clinical model, NRI = 0.2433, *p* = 0.0026, IDI = 0.1946, *p* = 0.00302; Test group: nomogram vs clinical model, NRI = 0.2105, *p* = 0.024, IDI = 0.1684, *p* = 0.028).

**Table 2:** Diagnostic efficiency of different models in the training and test cohorts

Model	AUC(95%CI)
Radiomics	
Training	0.95 (0.89–1.00)
Test	0.97 (0.91–1.00)
Clinics	
Training	0.89 (0.81–0.97)
Test	0.95 (0.87–1.00)
Nomogram	
Training	0.96 (0.91–1.00)
Test	0.98 (0.93–1.00)

*AUC* Area under the curve, *CI* Confidence intervals

**Fig. 7:**
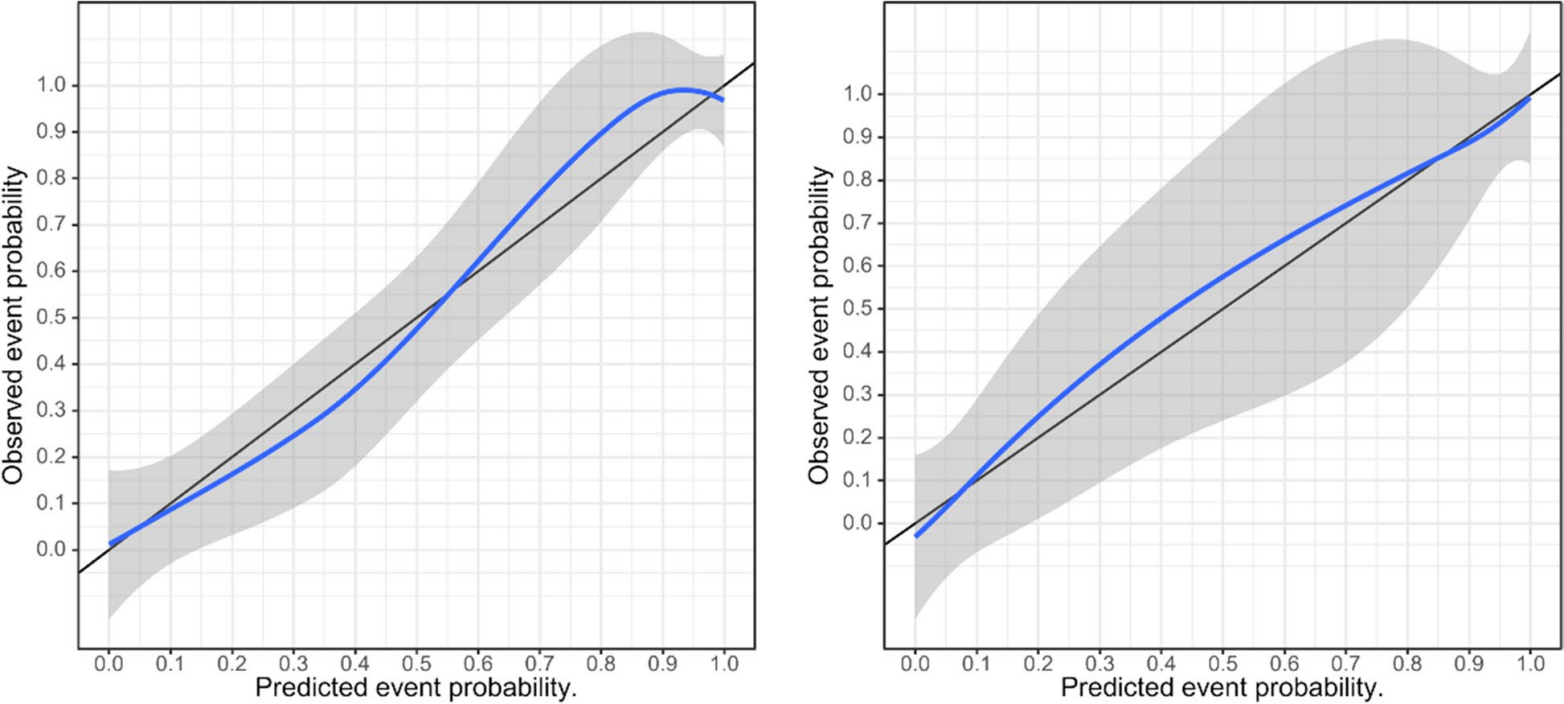
The calibration curve of the radiomics nomogram demonstrated good agreement between the predicted and expected probabilities for normal BMD and abnormally low BMD in both training (left) and test (right) cohort

**Fig. 8:**
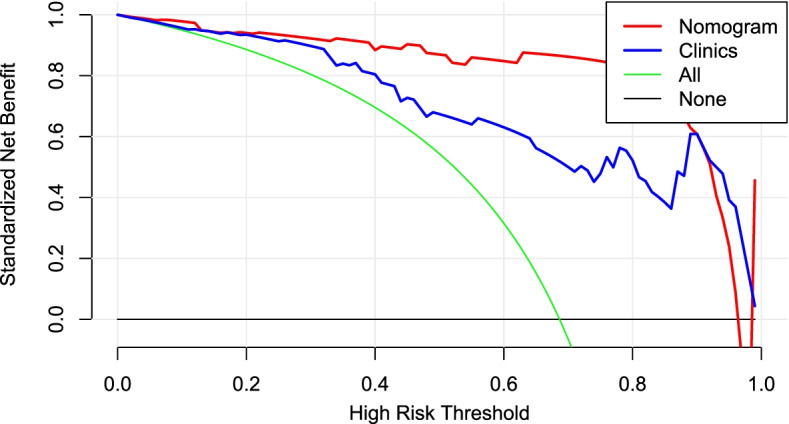
Decision curve analysis (DCA) for the radiomics nomogram. The DCA indicate that the application of radiomics nomogram to predict normal or abnomal BMD adds more benefit than clinical model. The red line and blue line represent the net benefit of the radiomics nomogram and the clinical model, respectively. The green line indicates the hypothesis that all patients had abnormal BMD. The black line represents the hypothesis that no patients had abnormal BMD

The calibration curve of the radiomics nomogram demonstrated good agreement between the predicted and expected probabilities for normal BMD and abnormally low BMD in training cohort, and *P* values of Hosmer-Lemeshow test were larger than 0.05 in both training and test cohorts (Fig. 7). Finally, we used decision curve to evaluate the clinical usefulness of the model (Fig. 8). It showed that the radiomics nomogram had a higher benefit in differentiating abnormally low BMD from normal BMD than the clinical factor model.

## Discussion

In the present study, we developed and validated a radiomics nomogram that incorporated one clinical factor and five radiomics features derived from fat-water imaging based dual-energy spectral CT, which can help clinician and radiologists to identify abnormally low BMD from normal BMD well. To the best of our knowledge, this is few radiomics model developed to diagnose osteoporosis combined with fat-water imaging based dual-energy spectral CT.

Osteoporosis is characterized by bone loss and increased susceptibility to fragility fractures [14]. Osteoporosis are associated with lower osteogenesis and greater adipogenesis, and the both arise from a common mesenchymal stem cell within bone marrow [15, 16]. The trabecular bone is the most metabolically active part in vertebrae [17]. Xiaojuan Li et al. found that the average fat content was significantly elevated of vertebral body in patients with osteoporosis/osteopenia compared with controls in 51 postmenopausal females by using magnetic resonance spectroscopy (MRS) [18]. Because DXA measures areal BMD, it can not differentiate between cortical and trabecular bone [19, 20], it doesn’t reflect fat content exactly as well.

Dual-energy CT measurements were used to derive basis material composition representation of the constituents of a measured volume. In a study by Bredella MA et al. [16], the L2 vertebra was scanned with dual-energy CT by using a dual source and multidetectors row CT scanner. They found excellent agreement between dual-energy CT and ^1^H MRS in the assessment of marrow adipose tissue (MAT) content of L2 vertebra. Different from their study, the dual-energy CT used in our study was based on a single tube fast switching between low-energy and high-energy within a rotation, which was called dual-energy spectral CT. In our study, the values from dual-energy spectral CT fat-water MD imaging of T11-L2 were measured, and there were significant differences between normal BMD and abnormal low BMD. This result showed that the values of fat-water MD can reflect the change of marrow adipose tissue of osteoporosis. Although the parameters extracted from ordinary CT images can also reflect osteoporosis, it can’t quantify bone mineralized component and bone marrow fat separately. To the best of our knowledge, there are few studies using fat-water MD images of dual-energy spectral CT as an evaluation method of osteoporosis.

Radiomics is a promising technique using computerized quantitative imaging analysis to extract a large number of image-related features to assist in diagnosing diseases, which has attracted increasing attention in recent years [21, 22]. In the present study, LASSO method was adopted to reduce the regression coefficient to construct the radiomics signature, which has already been used for the prediction of bone metastasis in prostate cancer [23] and colorectal cancer [24] patients. Finally, radscore was calculated by summing the selected five features weighted by their coefficients. The radiomics feature model based on fat-water imaging of dual-energy spectral CT showed sufficient discrimination in the training cohort (AUC = 0.95) and good predictive performance in the test cohort (AUC = 0.97). However, the radiomics model to predict abnormal BMD was not unique, for it was data-driven method, which might affect by the dataset and training method.

In the present study, five radiomics features consisted of the optimal feature subset. In these 5 features, 3 were the firstorder features and 2 were GLSZM features. And original_firstorder_90Percentile and wavelet_HLH_firstorder_Minimum contribute more to the radiomics model for they have larger coefficients. That meant that the grey value distribution within the ROI between the normal and low BMD were different. Besides, GLSZM zone percentage and Size Zone Nonuniformity also had contributions to radiomics model, and their coefficients were negative, which meant that the normal BMD had fine texture and more homogeneity. Gender and age were not statistically significant in the training and test cohort. Furthermore, these findings suggested that there were no differences of gender and age between abnormal and normal BMD. However, BMI was statistically significant in the test cohort only.

In addition to radiomics and clinics analysis, we also developed and evaluated the radiomics nomogram. The AUC value of radiomics nomogram achieved more satisfactory than radiomics and clinics model both in training and test cohort. The findings suggested that the radiomics nomogram based on the combined model had a higher benefit in differentiating abnormally low BMD from normal BMD. Moreover, DCA showed that employing the nomogram could obtain more net benefits than the clinical model alone. However, DCA is prevalence dependent, the result of DCA was only for reference. Thus, the use of the developed nomogram may be a promising method in assisting radiologists in differentiating abnormally low BMD from normal BMD. So as to help clinical early detection of osteoporosis and prevent the progress of osteoporosis.

The limitations of our study should be acknowledged. Firstly, it was a retrospective study performed in a single institution. Secondly, the number of patients enrolled in our study was relatively small. Additionally, there were a few clinics features included in present study, only age, gender, BMI and the values of fat-water imaging. In the future, the limitations need to be further improved in the following investigations.

## Conclusions

In conclusion, the present study developed a radiomics nomogram that incorporated clinical factor and radiomics features derived from fat-water imaging based dual-energy spectral CT, which may serve as an efficient tool to help clinician and radiologists to identify abnormally low BMD from normal BMD well.

## Data Availability

The datasets used and/or analyzed during the current study are available from the corresponding author on reasonable request.

## Abbreviations

AIC: Akaike an information
AUC: Area under the curve
BMD: Bone mineral density
BMI: Body mass index
CI: Confidence interval
CT: Computed tomography
DXA: Dual x-ray absorptiometry
LASSO: Least absolute shrinkage and selection operator
MAT: Marrow adipose tissue
MD: Material decomposition
mRMR: Minimum-Redundancy Maximum-Relevancy
PACS: Picture archiving and communication systems
ROC: Receiver-Operating-Characteristic
